# The Climate of the South of Devon, and Its Influence upon Health

**Published:** 1842-10

**Authors:** 


### Art. XIV.

*The Climate of the South of Devon; and its Influence upon Health.* By Thomas Shapter, M.D., &c.—*London*, 1842. 8vo. pp. 258.

The very admirable work, whose title we have just transcribed, and which, for its completeness, impartiality, and philosophical tone, may serve as a model for all future inquiries of the same nature, is in a great measure a reprint of papers already published in the Transactions of the Provincial Medical and Surgical Association, vols. vi, vii, and x. Such, however, is not its character throughout; many details have been omitted, others have undergone considerable condensation; the account of the geology of the district is rendered much more perfect, and there is an entirely new chapter, giving a short, but valuable sketch of the various towns and villages which are most commonly resorted to by invalids. We shall, therefore, treat it as a new book; and though, from the mass of materials, and, if we may so speak, the close-grained structure of the entire production, anything like a perfect analysis is impracticable; we shall endeavour to give a general outline of the volume, in as short a space as possible, advising such of our readers as desire further information, to procure and study the original for themselves.

The portion of the county noticed by Dr. Shapter, is the Southern district, including Exeter, and the well-known watering-places in its vicinity. Its general physiognomy is that of a succession of undulating high-grounds, with luxuriant vales, and small fertile valleys, for the most part in a state of high cultivation, and richly wooded by very lofty hedge rows; these, which are thickly studded with elm and oak trees, (the former, especially, in the vale of the Exe,) form boundaries of, generally speaking, very small inclosures. Besides the undulating high-grounds, there are hills and ridges of very considerable altitude, as Dartmoor, the mean height of which is 1782 feet, Haldon 800 feet, and Whitstone, 740 feet. The general beauty of the scenery is too well known to require notice. The *geological formation* of the district is very interesting, but can be barely touched upon within our limits. The series of rocks is extensive, ranging from the granite to the lower cretaceous group, but it is not complete in all its parts. The most important are, granite, grauwacke slate, carbonaceous rocks, schists, limestone, new red sandstone, (including *Exeter conglomerate*,) green sand, granite, green-stone, and trap-rocks. Coal, also, exists in one locality, Bovey heath, in conjunction with layers of Bovey clay. The coal is imperfectly formed, and is of little value as fuel.

The county abounds in rivers, streams, and springs. The chemical composition of the latter varies according to the geological formations in which they take their rise. Our author gives the analysis of several of each kind, for the particulars of which we must refer to the work itself (pp. 204-7); merely remarking, that where the mineral impregnations are in excess, which is particularly the case in some parts near the coast, as Torquay, it is advisable for invalids not to drink the water until it has been boiled, and thus freed of a portion of its solid ingredients, otherwise it is apt to occasion slight symptoms of a dyspeptic character. This observation, however, does not apply to those springs which are derived from the schist formation, the iron, and free carbonic acid, which many of them contain, rendering them, when drunk fresh, serviceable in cases of general debility and indigestion. The temperature of the springs is a little higher than the mean temperature of the climate.

The *flora* of South Devon is particularly rich; and the number of delicate exotics (see a list, p. 39) which flourish in the open air, and are not destroyed by exposure during the winter season, is of itself no mean proof of the mildness of the climate; to which subject we shall now direct our attention.

The most striking characteristic of the *climate* of Devon, generally, is that of being warm and moist; this is partly owing to its latitude, but also in a great measure to its position as regards the ocean, since it forms a portion of a large promontory, or imperfect peninsula, projecting westward into the Atlantic, and nearly half its circumference is sea coast. The mean annual temperature of South Devon, deduced from careful observations made at Exeter, is 51° 29′; this is nearly one degree higher than that of London, which is stated by Sir James Clark to be 50° 39′, though this is probably too high.

But it is not so much the actual warmth, as the equability of temperature, which characterizes the two south-western counties of England. The difference between the warmest and coldest of 10 years (from 1825 to 1834, both inclusive,) amounted, in South Devon, to no more than 4° (5° ?), and the mean difference, in succeeding years only to 1½°. The same relative superiority of equable temperature obtains, likewise, in the various months and seasons of each year, as is clearly manifested by Dr. Shapter's Tables, (p. 6,) in which the results of observation at Exeter are contrasted with records from the metropolis.

There is a curious circumstance connected with this subject, which we must notice, viz., that while the mean difference of temperature between day and night, for the whole year, amounts in this district to 30°, it is stated, by Luke Howard, not to exceed 14½° in London and the vicinity. This discrepancy obtains, likewise, in the mean diurnal variation which takes place during the several months and seasons. Dr. Shapter thinks the explanation of this singular anomaly must be sought, not only in the great quantity of heat artificially generated in London, " but also in the dense mass of houses radiating during the night the excess of temperature acquired during the day; the surrounding thick atmosphere constituting a medium which prevents this taking place as rapidly as if the sky were clear, and assisted by a free ventilation."

In South Devon, January is the coldest month; the temperature then progressively increases until July, when its mean height is 63°·64: after this it declines again to its minimum average 39°. The skies are, for the most part, disposed to be clouded and overcast, especially in January, December, November, February, March, and October; the above order indicating the relative proportion of shade proper to each month. April and May are the brightest and most sunshiny; July and August have only a moderate proportion.

The *hygrometrical constitution of the atmosphere* is a subject of much importance in the history of a climate, for slight variations in moisture are well known to exert a most powerful effect upon many forms of disease; and our author has, therefore, done wisely in devoting some space to its consideration. The only way in which the true condition of atmospheric moisture can be appreciated is by noting the difference between the temperature of the atmosphere, and the temperature at which dew is deposited; for when both are nearly the same, the climate is moist, and when they are widely separated, the climate is dry. Now, the results of a daily observation made in this way, at 9 A.M., for the space of five years (from 1832 to 1836 inclusive,) fully prove that the proverbial moisture of Devon is not imaginary; the mean temperature of the dew point being about 46° (45·9), while the temperature of the atmosphere itself is 53°, so that there is only a mean difference of four degrees and a half (4·4) between them. The winter season is the most damp, the differences of temperature amounting to but two degrees and a third (2·3); nor is the autumn much superior in this respect, the difference being very little more than three degrees (3·1.) The summer and spring are comparatively dry seasons, the mean difference amounting in the former to nearly eight degrees (7·9), and in the latter to nearly six (5·9). November is the dampest month, and July the dryest.

Common report gives Devon the character of being a particularly rainy county; but this opinion admits of some qualification. The annual fall of rain, at Exeter, amounts to very nearly 32 inches (31·90), being about 7 inches more than in London. But, notwithstanding this, the number of wet days is not so great, the average for Exeter being 162·4, while in London it is 178. By a *wet day* is understood one on which a fall of rain, however slight, takes place. It should also be observed that some places on the coast are both less liable to rain and fog, and have generally a dryer atmosphere than Exeter and other parts of the district. This is especially the case with Torquay, Exmouth, and Budleigh Salterton (vide pp. 142, 153.)

*Frost* is not uncommon in this district, but the atmosphere very rarely maintains, for any length of time, a temperature below the freezing point. *Snow* seldom falls in any great quantity, or remains upon the ground above two or three days. The fall nearly always takes place in January and February. *Hail* is a little more frequent than snow. In ten years the number of days in which it fell amounted to 71. It is most prevalent in April and December. *Thunder* and *lightning* are, comparatively, unfrequent, and the storms are very rarely attended with serious consequences.

The most prevailing *winds* of this district are the w. and n. w. They occur chiefly in June, November, February, December, and March. The e. and s. e. prevail most in May, July, and October. s. and s. w. in September, and n. and n. e. in January. Taking the average of the whole year, the s. and s. w. are attended by the highest temperature, and the n. and n. e. by the lowest: but this does not apply to individual months, for in June the n. and n. e. are the warmest winds, and the same is also true of the e. and s. e. in May. During the winter season the s. w. wind is often accompanied by a warm thick mist, which is peculiarly relaxing, and from its frequency is not unaptly styled Devonshire weather. A similar phenomenon occasionally attends the s. e. wind during summer. We must refer to the work itself for a number of tables illustrating these various points (pp. 31, 32.)

Upon the whole, then, it would appear "that the chief characteristic of the climate of this district is that of being warm, soft, mild, equable, calm, and free from storms; though subject to a large share of rain, yet it seldom occurs that a whole day is so unceasingly wet, as not to afford some hours for out-door exercise..... The air is usually damp, but from the general prevalence of warm westerly winds, the moisture which it contains is not cold and chilling."

The most important part of Dr. Shapter's volume is that in which the effects of the climate upon the constitutions of its inhabitants are illustrated. The data, from which the results are deduced, are the cases treated, during the same ten years over which the former observations extend, at the Exeter Dispensary: and amounting as they do to no less a number than 11,258, (4535 males and 6723 females,) the inferences deduced are worthy of all confidence.

We would gladly transfer to our pages one of the tables given by our author, (No. iii,) showing the relative frequency of various diseases in each month, but could not do so without occupying more space than can well be spared, and must therefore content ourselves with a short abstract of the results. From this table it appears, that the more sickly period of the year extends from February to July, but that the greatest amount of sickness prevails during April and May: and the climate of these two months is characterized by some peculiarities which are worthy of notice. It is between them that the rise in temperature is most considerable, amounting to more than one half of the whole difference between spring and summer; the difference between the diurnal maximum and minimum is also more marked than in any of the other months, amounting in April to nearly 39°, and in May to 34½°, 30° being the mean during the year. The mean barometric height is lower than in either of the two months, which immediately precede or succeed them; the most prevalent winds are the N. N. E., and S. E., and the number of sunshiny days is greater than during other parts of the year. This sickly period is, therefore, characterized by a sudden increase of temperature, by warm days and cold nights, by a dry atmosphere, and seductive sunshine; and the diseases which make up the increase are, in April, fever, rheumatism, dropsy, diseases of the brain, bronchitis and pneumonia, dyspepsia, gastritis, and diseases peculiar to females;—in May, eruptive fevers, scrofula, diseases of the brain and heart, bronchitis, dyspepsia, and eruptive complaints. It should be observed, however, that the mortality in these same months is rather below the average, as we shall see presently.

The period of the year in which there is the least amount of disease extends from August to January, September and October being the most healthy months. These two months are characterized by equability of temperature, the mean diurnal difference being no more than 29°. The prevailing winds are from the west; the air is moist when the temperature is high, and dry when it is low; and there is a good deal of rain. This bears out the statement made above, that the thick, warm, and commonly-styled unwholesome weather, is in reality conducive to health in this district. The diseases which more particularly diminish in frequency during this period are dropsy, diseases of the chest and brain, those peculiar to females, and rheumatism; fever, on the contrary, begins to increase.

*Fever* constitutes 12 per cent. of the whole amount of disease in the 11,258 cases observed. This average, though large in itself, is lower than that found in other great towns, as London, Edinburgh, Glasgow, &c. Its frequency, of course, varies in different years. The greatest number of cases, during the ten years, occurred in 1827, but there was nothing peculiar in the climate to account for this increase. February appears to be the month most prone to fever, and the fewest cases occur in August and September. The average age of the patients was 31; this is below the average given by Dr. Craigie,* which was 45, and above that recorded by Dr. Tweedie,† who observed the greatest number of cases between 20 and 25. The most common types are simple *synochus* and *typhus*. In most cases some degree of local inflammation has been noticed with the general fever; the adynamic typhus being most frequently complicated with affections of the mucous membrane of the stomach and bowels, then of the viscera of the chest, and more rarely of the brain, (this latter assertion is only true of the early stages.) In the other, and

* Edinb. Med. and Surg. Journal, vol. xlvi. † Cyclop. of Pract. Med., vol. ii. p. 189.

more simple fevers of the district, the chest is most frequently attacked, then the abdomen, and then the brain. It would not appear that these diseases are accompanied by a high degree of mortality. Dr. Shapter inclines to the opinion that fever sometimes originates tuberculous deposits, in patients who are predisposed to them (p. 59). The peculiar eruptions of fever are not, generally speaking, of frequent occurrence, though at times they prevail extensively, the most usual form being that of petechiæ. In 1836 a peculiar form of *spotted fever* was epidemic. The spots appeared within the first twenty-four hours. They were of a brownish red, sometimes passing into a purplish colour, larger than petechiæ, and gradually shaded off into the deepened colour of the surrounding integuments. Some of them passed into vesicles, and they generally terminated by desquamation of the cuticle. The fever was of an adynamic character, and was most successfully treated with salines and aromatics. In 1839 and 1840, fever assumed a hemorrhagic tendency, and proved fatal in many cases, by bloody discharges from the bowels. Twice or three times in the last ten years the external mucous surfaces in children, during a scarcely appreciable attack of fever, showed a great liability to take on inflammation of a bad character, attended by copious muco-puriform discharges. In 1834 this was peculiarly the case with the female organs of generation, and in an ignorance of the circumstances suspicion might have been excited that disease had been communicated, attended with violence.

*Intermittent fever* is of very rare occurrence. The ordinary *eruptive* fevers occur in this district in about their usual proportion, generally assuming an epidemic character. With reference to these we have little to notice, excepting that in cases of *malignant scarlatina*, in which all other plans of treatment entirely failed, Dr. Shapter has obtained the most signal benefit from the free exhibition of ammonia, according to Dr. Peart's method. "Not only," he says, "were present symptoms relieved, and danger almost invariably arrested by its use, but I observed that the patients stepped, as it were, immediately from the sick bed into health; there was nothing like a protracted convalescence," and disagreeable sequelæ were far from frequent.

*Rheumatism*, *lumbago*, *gout*, &c., though not infrequent disorders, are by no means so prevalent as in many other parts of the kingdom—the number of cases forming about 4 per cent., or 1 in 24 of the whole registered diseases; while in Penzance they constitute 1 in 17·6; in Plymouth, 1 in 18·4; in London, 1 in 14·7; and in the north of England, 1 in 22. The cases which occur consist chiefly of subacute rheumatism, rheumatic gout, and more especially chronic rheumatism. Acute rheumatism is only occasionally met with. These various forms are most prevalent in March, April, May, and June.

*Dropsy* is more frequent in this district than in many other places, forming $3\frac{1}{2}$ per cent., or 1 in 28 of the whole diseases. How far this may depend upon the moisture of the climate, is a question we do not feel ourselves competent to answer.

*Diabetes mellitus* and *insipidus* are by no means uncommon. They occur chiefly in males. Every case of true diabetes which has come under Dr. Shapter's notice, terminated in tubercular phthisis. "This has been so uniformly the case," he says, "that the conviction forces itself upon me of its being essentially a scrofulous disease, and that the kidney is made to be an emunctory of those matters otherwise colliquatively discharged by the skin."

*Scrofula* is more frequent than in the midland counties; it constitutes nearly 2 per cent. of the whole number of cases. It appears in all its various forms. Mr. James, of Exeter, has treated *noli me tangere* with the most complete success, by a compound of the chloride of zinc (1 part) with sulphate of lime (3 parts). This is moistened, and applied on lint over a small portion of the diseased surface; after about five days it falls off, exposing a healthy-looking sore, which readily heals on the application of simple dressing. Other portions are then similarly treated, until the cure is complete.

*Scirrhus* occurs in about the usual average, i. e. about 1 in 244.

We pass over the diseases of the brain and nerves, and also those of the heart and great vessels, which present nothing to detain us, and proceed to consider—*Diseases of the lungs*, which form 17 per cent. or 1 in 6 of the whole amount. Of these a large proportion consists of *simple bronchitis*, which is most prevalent during the winter and spring months. *Chronic catarrh* is not uncommon among the lower orders, but is comparatively rare among the upper classes. *Influenza* has been epidemic several times, and proved, as in other places, very fatal. *Asthma* is rarely met with. *Croup* is at times frequent, generally making its appearance as an epidemic. *Laryngismus stridulus* also occasionally occurs; and so does *hooping-cough*, but only as an epidemic. *Consumption* forms a large proportion (4 per cent.) of the diseases of the chest in this district; it occurs in all its various forms, though, most usually, the cases are protracted and lingering. *Pneumonia* is by no means frequent. *Pleuritis* is more common. *Diseases of the abdomen* amount to $14\frac{1}{4}$ per cent. of the whole. *Dysentery* is rare: *diarrhœa* is very prevalent, especially among children. *Acute gastritis* seldom occurs; the *chronic* form is more frequent. *Peritonitis* was very common in 1827. The *lead*, or as it has been called, the *Devonshire colic*, has disappeared (excepting among painters, &c.), with the removal of its cause, viz., the employment of leaden vessels in the making of cider. *Dyspepsia* prevails to a considerable extent, but presents no remarkable features. The largest proportion of cases takes place in May. *Functional disorders of the liver* are not infrequent; but what is generally understood by the term "*liver disease*," is by no means common. Diseases of the *urinary organs* are not frequent. *Uterine diseases* occur in about the ordinary proportion. *Eruptive diseases* form a considerable proportion of the disorders of the district, about 1 in 14. The most common are scabies, herpes, impetigo, psoriasis, lepra, eczema, prurigo, and acne.

Having given a summary of the indigenous diseases our author proceeds, in the next chapter, to specify those affections in which a change to the climate of South Devon appears beneficial. Here we shall follow him, with all possible brevity. Much advantage is frequently derived from a residence in this climate, at those periods of life when the system undergoes changes, which, without the presence of any active disease, often give rise to so much weakness and depression as to cause great anxiety in the minds both of the patient and his friends. This is especially the case in early youth, when the growth of the body is disproportioned to the strength,—at the commencement of decay, when various symptoms arise, to which the name of *climateric disease* has been applied,—and in the general failing of advanced years. In all these cases, a change to this locality is often marked by a speedy alleviation of distress. It is also to be particularly recommended in those cases where a tendency to scrofulous disease is manifested, but not to the exclusion of other dietetic and medicinal preventive means, as is so frequently supposed by parents and patients.

As a residence for consumptive patients the southern parts of Devonshire have long been celebrated. Dr. Shapter speaks on this subject with the reserve of a cautious and experienced physician. "This district," he says, " possesses many of the qualifications which are usually thought requisite for counteracting the consumptive tendency, such as, its contiguity to the sea ; its protective amphitheatre of hills, forming an adequate barrier against the north and east winds ; together with an atmosphere soft, warm, and charged with aqueous moisture, a condition eminently useful towards alleviating irritation of the lungs." (p. 122.) Torquay and Dawlish are the places most recommended. Of the precise character of the climate of the former place Dr. Shapter speaks doubtingly, notwithstanding the numerous observations on its temperature, &c., that have been put on record. Dr. S. thinks these observations have been made " at such irregular times, and for so short a period at each time, that it is out of the question deducing from them anything like satisfactory averages." There can be little doubt, however, that of all the places on the south-western coast, Torquay affords the driest and most sheltered position during the winter months.

The climate of South Devon is also calculated to give much relief in *bronchitic affections ;* in *dyspepsia,* unless associated with, or depending upon, general debility ; in *gout* and *rheumatism ;* in *dysentery,* and in *cutaneous affections.* It is, moreover, very useful in that form of *amenorrhœa* which is characterized by the bloodless cheek and lip, shortness of breath, nervous palpitation, quick small pulse, pain referred to a spot beneath the left breast, and general tendency to constipation.

The next four chapters contain accounts of the different watering-places, the geology and natural productions of the district (already considered by us), and the civil and economical history of the inhabitants ; and the last, and certainly not the least important section of the work, gives the vital statistics of Exeter and the immediate neighbourhood.

Of this last division of the work we regret that our limits will not allow us to give any account ; but we can safely recommend it as a model for those engaged in similar inquiries. The data, of the most authentic kind, have been weighed with the most scrupulous accuracy, and the results, therefore, may be thoroughly depended on.

In concluding this very imperfect sketch of Dr. Shapter's valuable and interesting work, we must once more recommend it to the attention of our readers. Independently of the important information of various kinds contained in it, it may safely be studied as a model for those who are desirous of pursuing a similar line of inquiry, and who wish to see the medical topography of a district treated with that singleness of purpose, and philosophical candour, which should characterize the writings of every member of a liberal profession.

